# A Single Sauna Session Does Not Improve Postprandial Blood Glucose Handling in Individuals with Type 2 Diabetes Mellitus: A Cross-Over, Randomized, Controlled Trial

**DOI:** 10.1055/a-2406-4491

**Published:** 2024-09-26

**Authors:** Laura Schenaarts, Floris K Hendriks, Cas J Fuchs, Wendy EM Sluijsmans, Tim Snijders, Luc JC van Loon

**Affiliations:** 1 385783 Department of Human Biology, Maastricht University School of Nutrition and Translational Research in Metabolism, Maastricht, Netherlands

**Keywords:** passive heating, glucose tolerance test, insulin sensitivity, glucose metabolism, thermoregulation

## Abstract

**Introduction** Passive heat treatment has been suggested to improve glycemic control in individuals with type 2 diabetes mellitus (T2DM). Previous studies have focused predominantly on hot water immersion and traditional sauna bathing, as opposed to the more novel method of infrared-based sauna bathing. Here, the impact of a single infrared sauna session on post-prandial glycemic control was assessed in older individuals with T2DM.

**Methods** In this randomized controlled crossover trial, 12 participants with T2DM (male/female: 10/2, age: 69±7 y, BMI: 27.5±2.9 kg/m ^2^ ) rested in an infrared sauna twice: once in a heated (60°C) and once in a thermoneutral (21°C) condition for 40 min, immediately followed by a 2-h oral glucose tolerance test (OGTT). Venous blood samples were obtained to assess plasma glucose and insulin concentrations and to determine the whole-body composite insulin sensitivity index.

**Results** Body core and leg skin temperature were higher following the heated condition compared to the thermoneutral condition (38.0±0.3 vs. 36.6±0.2°C and 39.4±0.8 vs. 31.3±0.8°C, respectively; P<0.001 for both). The incremental area under the curve (iAUC) of plasma glucose concentrations during the OGTT was higher after the heated condition compared to the thermoneutral condition (17.7±3.1 vs. 14.8±2.8 mmol/L/120 min; P<0.001). No differences were observed in plasma insulin concentrations (heated: 380±194 vs. thermoneutral: 376±210 pmol/L/120 min; P=0.93) or whole-body composite insulin sensitivity indexes (4.5±2.8 vs. 4.5±2.1; P=0.67).

**Conclusions** A single infrared sauna session does not improve postprandial blood glucose handling in individuals with T2DM. Future studies should assess the effect of more prolonged application of infrared sauna bathing on daily glycemic control.

## Introduction

Type 2 diabetes mellitus (T2DM) is a metabolic disorder characterized by poor glycemic control. Prolonged postprandial hyperglycemia is a major risk factor for the development of microvascular complications and cardiovascular disease 1 . Nearly 45% of individuals with T2DM fail to achieve recommended blood glucose levels (HbA1c<53 mmol HbA1c/mol Hb) 2 . Though lifestyle-based regimens can effectively improve glycemic control in T2DM 3 , adherence to such regimens is generally low 4 5 . Therefore, additional strategies are required to improve glycemic control in individuals with T2DM.

Passive heating has been proposed as a potential additional approach due to some similarities with low-intensity exercise 6 . This is interesting in the context of T2DM, considering that exercise is a well-established non-pharmacological intervention known to improve glycemic control in this population 3 7 . Physiological changes induced by physical exercise, such as increased heart rate, body temperature, sweat rates, and peripheral blood flow, are also observed during heat stress 8 9 . These thermoregulatory responses are crucial for maintaining physiological core temperatures in acute heat stress 8 10 . Despite previous assumptions that thermoregulatory vasodilation exclusively occurs cutaneously 11 , heat stress also induces a concomitant elevation in skeletal muscle blood flow 9 12 13 . As this may promote insulin-mediated glucose uptake in skeletal muscle tissue 14 , passive heat treatment can potentially enhance postprandial peripheral glucose uptake in individuals with T2DM.

Despite its theoretical benefits, studies using a single session of hot water immersion as the passive heat modality have so far failed to observe any improvements in postprandial glucose concentrations and insulin sensitivity in individuals with T2DM 15 16 17 . Given that different heating modalities have shown distinct effects on physiological outcomes (e. g., body temperature) 18 19 highlights the importance of evaluating different passive heat modalities on health-related outcomes 20 . Unlike hot water immersion, in which heat is transferred through the skin, an infrared sauna utilizes infrared waves that penetrate beyond the superficial layers of the skin 21 , thereby facilitating a more targeted heating effect of deeper tissues. Hence, this approach may augment the temperature of peripheral skeletal muscles and improve muscle perfusion more effectively. As a result, an infrared sauna could be a beneficial heat treatment modality to improve peripheral glucose uptake in individuals with T2DM. To date, no studies have assessed how infrared sauna bathing acutely affects postprandial glycemic excursions in individuals with T2DM.

This study investigated the impact of a single infrared sauna bathing session on glycemic excursions during a subsequent oral glucose tolerance test (OGTT) in individuals with T2DM. We hypothesized that, compared to a thermoneutral control condition, infrared sauna bathing lowers postprandial glycemic excursions in individuals with T2DM.

## Methods

### Participants

Twelve men and women with T2DM, aged 50 y or older and using at least one oral hypoglycemic agent, were recruited to participate in the current study through advertisements ( Table 1 ). Exclusion criteria included insulin usage, recent changes in diabetes medication, frequent (≥1 time/week) use of sauna, participation in a structured exercise program in the past 3 months,>5% weight change over the past 6 months, inability to tolerate high temperatures, smoking, and diagnosis of medical condition(s) that could jeopardize participant safety or hinder data interpretation.

**Table:** **Table 1** Participant characteristics.

	Participants ( *n=* 12)
Age (y)	69±7
Sex	
Female	2
Male	10
Body mass (kg)	83.4±12.2
Height (m)	1.74±0.10
BMI (kg/m ^2^ )	27.5±2.9
Fat free mass (kg)	33.0±5.4
Fat mass (kg)	23.8±5.9
Fat percentage (%)	28.5±4.9
Time since onset T2DM (y)	12±7
HbA1c (mmol HbA1c/mol Hb)	55.0±7.1
HOMA-IR	2.4±1.3
Number of hypoglycemic agents	2.0±0.6
Metformin	11
SGLT2-inhibitors	3
DPP4-inhibitors	3
Sulfonylureum derivates	7

Values are expressed as means±SDs. BMI: body mass index. HbA1c: Hemoglobin A1c. T2DM: type 2 diabetes mellitus. HOMA-IR: Homeostatis Model Assessment for Insulin Resistance; SGLT2: sodium-glucose cotransporter-2; DPP4: dipeptidyl peptidase 4.

All participants were fully informed regarding the experimental procedures and associated risks. The remaining queries were answered before written informed consent was obtained. The study was conducted in accordance with the principles outlined in the Declaration of Helsinki and was approved by the Medical Research Ethics Committee Academic Hospital Maastricht/Maastricht University (METC 22–057). The study was registered on ClinicalTrials.gov (NCT05610046).

### Study design

This counterbalanced randomized cross-over controlled trial was conducted at the Department of Human Biology, Maastricht University, The Netherlands, between February 2023 and April 2023 to minimize natural heat acclimatization. Participants were randomly allocated to start with the HOT (infrared sauna at 60°C) or CON (thermoneutral) experimental condition by an independent researcher according to a block randomization plan using an online block randomizer ( http://www.randomization.com ). Participants underwent the HOT and CON conditions in a counterbalanced order: at rest in a seated position in an infrared sauna (HM-LSE-3 Professional edition, Health Mate, Belgium) at 60°C (humidity is not controlled in an infrared sauna) for 40 min (HOT) and in thermoneutral conditions at 21°C for 40 min (CON), immediately followed by a 7-point OGTT to determine postprandial glycemic excursions. Outcome parameters included plasma glucose and insulin concentrations, tympanic and skin temperature, hematocrit, blood pressure, and heart rate. Experimental trials were completed with a wash-out period of at least 7 days between visits to limit acclimation effects. Humidity in the laboratory was 64±2.7%.

### Screening

All participants were invited to the laboratory for a screening visit to assess eligibility for the study. Participants arrived in the laboratory in a fasted state and without consumption of their morning hypoglycemic medication. Medical history was assessed, and heart and lungs were auscultated by a qualified physician. Subsequently, body mass and height, resting blood pressure, and resting heart rate were measured. A multi-frequency bioelectrical impedance analyzer (InBody-S10; Biospace, Cerritos, CA, USA) was used according to the manufacturer’s guidelines for the estimation of body composition. Lastly, all participants were familiarized with infrared sauna bathing at 60°C for 30 min.

### Instructions prior to test days

Throughout the 2 days prior to the experimental procedures, participants were instructed to maintain their habitual diet and activity levels, refrain from strenuous physical activities, and avoid alcohol consumption. A standardized meal (~3000 kJ, providing 54 energy percent (En%) carbohydrates, 27 En% fat, and 16 En% protein) was consumed before 10:00 PM prior to each test day, and followed by an overnight fast. On the morning of each test day, participants were asked not to consume any hypoglycemic medication. Food intake was recorded in 2-day food diaries before both test days to allow replication and check for differences between test days. Food intake records were summarized based on the Dutch food composition table NEVO 2021 22 and described as total energy intake (kJ), total carbohydrate, total protein, and total fat intake (En%).

### Experimental visits

**Fig. 1:**
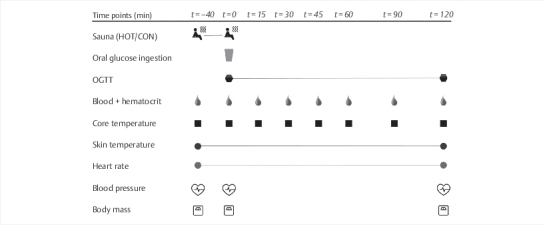
Schematic representation of an experimental visit. CON, control: 40 min at 21°C; HOT, infrared sauna: 40 min at 60°C; OGTT, oral glucose tolerance test.

An overview of the experimental protocol is depicted in Fig. 1 . Participants reported to the laboratory between 08:00 and 09:00 AM, after which resting blood pressure, body mass, and tympanic temperature (as a measure for core temperature) were measured. Subsequently, a heart rate monitor was adjusted around the chest and skin thermometers (iButton, Maxim Integrated Products, San Jose, CA, USA) were applied on both upper legs to continuously measure heart rate and skin temperature, respectively, throughout the test day. Tympanic temperature was measured using a tympanic thermometer (Braun ThermoScan IRT 6520, Kronberg, Germany) with each blood collection. After baseline measurements, a cannula was inserted into an antecubital vein. Following pre-sauna blood sampling (baseline, t=− 40 min), participants entered the infrared sauna and remained seated for 40 min in the HOT or CON condition, wearing underwear only. Directly after exiting the sauna, tympanic temperature and blood pressure were measured. After removing all sweat from the body, body mass was determined. A blood sample (t=0 min) was collected before consumption of the glucose beverage containing 75 g glucose dissolved in 200 mL water (LemonGluc, Novolab, Belgium), after which participants were allowed to drink 100 mL of water. The 2-h OGTT commenced within 10 min after exiting the sauna. During the OGTT, participants remained seated (were allowed to read) and wore clothing of choice. Additional blood samples were obtained at t=15, 30, 45, 60, 90, and 120 min. Blood pressure and body mass were measured again following the final blood draw.

### Blood analysis

On the first test day, one blood sample was collected at baseline in a heparin-containing tube for HbA1c analysis, while all other blood samples were collected in EDTA-containing tubes. Homogenized blood was collected into three heparinized micro-hematocrit capillary tubes, centrifuged (5 min at 8500 *g* at 21°C) and subsequently, hematocrit values were determined. Thereafter, the remaining blood was centrifuged (10 min at 1000 *g* at 4°C) to obtain plasma. Aliquots of plasma were frozen in liquid nitrogen and stored at − 80°C until analysis of plasma glucose, insulin, noradrenaline, and cortisol concentrations using commercially available kits (glucose HK CP, ABX Diagnostics, Montpellier, France; Human Insulin ELISA, Meso Scale Discovery, Rockville, Maryland, USA; TECAN ELISA, IBL International GmbH, Hamburg, Germany, respectively).

### Insulin sensitivity indices

Plasma glucose and insulin concentrations throughout the 7-point OGTT were used to calculate the whole-body composite insulin sensitivity index (ISI _composite_ ) as defined by Matsuda and DeFronzo 23 , as shown in the formula below:



where G _0_ and I _0_ represent fasting post-sauna (t=0 min) plasma glucose (mg/dL) and insulin (mUi/L) concentrations, respectively. G _mean_ and I _mean_ represent time-weighted means of plasma glucose and insulin concentrations during the OGTT. Additionally, tissue-specific insulin sensitivity indices including the hepatic insulin resistance index (HIRI) and muscle insulin resistance index (MISI) 24 were calculated. HIRI and MISI were calculated using the formulas as shown below:





The incremental area under the curve (iAUC) for plasma glucose and insulin concentrations during the OGTT were calculated based on the trapezoid method, using fasting post-sauna plasma glucose (mmol/L) and insulin (pmol/L) concentrations as a baseline. The Homeostatis Model Assessment for Insulin Resistance (HOMA-IR) 25 was determined from fasting pre-sauna plasma glucose (mg/dL) and insulin (mUi/L) concentrations.

### Statistics

All data were tested for normality using the Shapiro-Wilk test ( *P<* 0.05). Wilcoxon Signed-Rank test was used for non-normally distributed data of ISI _composite,_ HIRI and MISI. A paired samples T-test was used to analyze the iAUC of plasma glucose and insulin. Time-dependent outcomes (i. e., plasma glucose and insulin concentrations, noradrenaline, and cortisol concentrations, hematocrit, tympanic and skin temperature, heart rate, and blood pressure) were analyzed using two-way (time×condition) repeated measures analysis of variance (ANOVA). If a significant time effect was observed, Bonferroni post-hoc corrections were applied to localize differences between time-points. In case of a significant interaction, separate one-way repeated measures ANOVA analyses were performed for HOT and CON to locate significant differences between time-points and paired samples T-tests were used to analyze differences between HOT and CON at all time-points. T-tests were not corrected for multiple comparisons. Correlations were explored between plasma noradrenaline and cortisol concentrations and iAUC of plasma glucose and insulin during HOT and CON for time points t=− 40, t=0, and t=60 min using Pearson’s correlation coefficients. Significance was set at *P* <0.05. Normally distributed data are presented as means±SDs and not normally distributed data as medians with [95% confidence intervals]. Statistical analyses were performed using the statistical software program IBM SPSS (version 28.0, IBM Corp., Armonk, NY, USA).

## Results

### Participant characteristics

Participant characteristics are depicted in Table 1 **.** All participants completed both test days. No adverse effects were reported.

### Thermoregulatory response

**Fig. 2:**
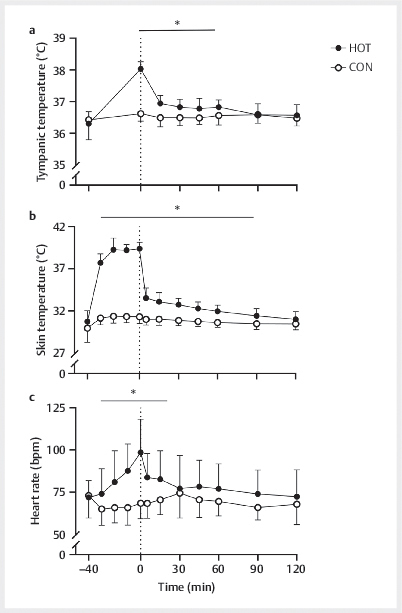
Tympanic temperature ( **a** ), skin temperature ( **b** ), and heart rate ( **c** ) throughout the experimental visit. The glucose beverage was ingested at t=0 min (dotted line). Data are presented as means±SDs, n=12. *HOT is significantly higher than CON ( *P* <0.05). Data were analysed using two-way (time×condition) repeated measures ANOVAs. CON, control: 40 min at 21°C; HOT, infrared sauna: 40 min at 60°C.

Tympanic and skin temperature were not different at baseline in HOT ( *P=* 0.384) and CON ( *P=* 0.157), Fig. 2a–b . A significant time×condition interaction was observed for tympanic and skin temperature ( *P<* 0.001 for both). Between baseline and t=0 min (pre- to post-sauna) in HOT, the tympanic temperature increased ( *P* <0.001). Likewise, skin temperature in HOT increased from baseline to t=0 min ( *P<* 0.001), while no changes in tympanic and skin temperature were observed over time for CON ( *P* >0.05). At t=0 min, tympanic and skin temperature were higher in HOT compared to CON ( *P* <0.001 for both). Compared to CON, the tympanic temperature in HOT remained elevated until t=60 min ( *P<* 0.05 for all), while skin temperature remained higher until t=90 min ( *P* <0.05 for all).

### Plasma glucose and insulin concentrations

**Fig. 3 FI02-2024-0041-dia-0003:**
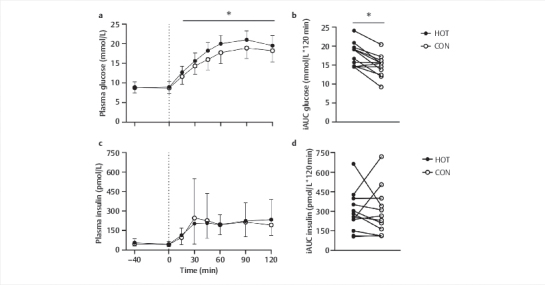
Plasma glucose ( **a** ) and insulin ( **c** ) concentrations throughout the experimental visit and iAUC for glucose ( **b** ) and insulin ( **d** ) dext-linkng the OGTT. The glucose beverage was ingested at t=0 min (dotted line). Data are presented as means±SDs including individuals data points for **c** and **d** , n=12. *HOT is significantly higher than CON ( *P* <0.05). Data were analysed using two-way (time×condition) repeated measures ANOVAs for panel A and C and using paired T-tests for panel **b** and **d** . CON, control: 40 min at 21°C; HOT, infrared sauna: 40 min at 60°C; iAUC, incremental area under the curve; OGTT, oral glucose tolerance test.

Plasma glucose concentrations at baseline were not different between HOT and CON ( *P=* 0.81, Fig. 3a ) *.* Plasma glucose concentrations did not differ between baseline and t=0 min in HOT ( *P* =1.0) and CON ( *P* =1.0). A significant time×condition interaction was found for plasma glucose concentrations throughout the test day ( *P* <0.001). Plasma glucose concentrations increased following glucose ingestion and remained elevated until the end of the test day in both conditions ( *P* <0.001 for all values after t=0 min). From t=15 to t=120 min, plasma glucose concentrations were higher in HOT compared to CON ( *P* <0.05 for all). The iAUC of plasma glucose concentrations ( *P* <0.001) and peak plasma glucose concentrations ( *P* <0.001) were also higher in HOT when compared to CON. A significant effect of time ( *P* <0.001), but not condition ( *P* =0.92) was observed for plasma insulin concentrations throughout the test days ( Fig. 3c ). Compared to t=0 min, plasma insulin concentrations were significantly higher at t=15, 45, 60, 90, and 120 min in HOT ( *P<* 0.05 for all) and t=15, 60, 90, and 120 min in CON ( *P* <0.05 for all), with no difference between the two groups (time×condition interaction, *P=* 0.059). Insulin iAUC did not differ between conditions ( *P* =0.93). Hematocrit at baseline was 43.0±3.1 and 42.5±3.6% during HOT and CON, respectively, and did not differ between conditions or over time (time: *P=* 0.60; condition: *P=* 0.60; time×condition: *P* =0.15). Similar outcomes were observed when plasma glucose and insulin values were corrected for hematocrit ( **Supplementary Figure 1** ).

### Insulin sensitivity indices

**Fig. 4:**
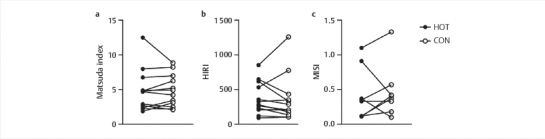
Insulin sensitivity indices. Matsuda index, n=12 ( **a** ) HIRI, n=12 ( **b** ) MISI, n=8 ( **c** ) for HOT and CON, derived from plasma glucose and insulin concentrations dext-linkng the 2-h OGTT. Data are presented as individuals data points. Data were analysed using non-parametric Wilcoxon Signed-Rank test. HIRI: hepatic insulin resistance index; Matsuda index: whole-body composite insulin sensitivity index; MISI: muscle insulin sensitivity index.

No differences were observed between HOT and CON for ISI _composite_ ( *P* =0.67), HIRI ( *P* =0.39), and MISI ( *P* =0.73), as depicted in Fig. 4 .

### Cardiovascular response

Heart rate was not recorded for *n=* 1 during CON due to technical malfunctioning of the heart rate monitor. Heart rate did not differ at baseline between conditions ( *P=* 0.63, Fig. 2c ). A significant time×condition interaction was found for heart rate ( *P<* 0.001). Heart rate increased between baseline and t=0 min in HOT ( *P=* 0.04), while no changes over time were observed for CON ( *P=* 1.0). Heart rate was higher in HOT than in CON between t=− 30 and t=15 min ( *P<* 0.05 for all).

Systolic blood pressure (SBP) at baseline was not different in HOT and CON (144±13 and 144±13 mmHg; *P* =1.0, **Supplementary figure 2** ). A main effect of time ( *P* =0.005), but not condition ( *P* =0.139) was observed for SBP. SBP decreased from 144±13 at baseline to 133±19 mmHg at t=0 min during both HOT and CON ( *P=* 0.008), with no difference between the two groups (time×condition interaction, *P* =0.222). Diastolic blood pressure (DBP) was not different between HOT and CON at baseline (78±6 vs. 79±7 mmHg; *P=* 0.61). DBP showed a significant time×condition interaction ( *P* =0.013). During HOT, DBP decreased from 78±6 to 63±11 mmHg between baseline and t=0 ( *P* <0.001) and did not return to baseline at t=120 (72±11 mmHg; *P* =0.011), while no changes over time occurred during CON. DPB was lower in HOT compared to CON at t=0 min (63±11 vs. 77±12 mmHg; *P* =0.005) and t=120 min (72±11 vs. 79±14 mmHg; *P* =0.021).

### Catecholamines

**Fig. 5:**
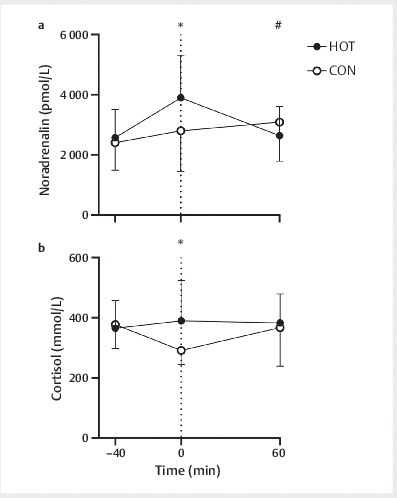
Noradrenaline ( **a** ) and cortisol ( **b** ) concentrations throughout the experiment at t=− 40, 0, and 60 min. The glucose beverage was ingested at t=0 min (dotted line). Data are presented as means±SDs, n=12. *HOT is significantly higher than CON ( *P* <0.05). Data were analysed using two-way (time×condition) repeated measures ANOVAs. CON, control: 40 min at 21°C; HOT, infrared sauna: 40 min at 60°C.

Noradrenaline and cortisol concentrations did not differ between HOT and CON at baseline ( *P* >0.05, Fig. 5 ). A significant time×condition interaction was found for both noradrenaline and cortisol concentrations ( *P* <0.001). In HOT, noradrenaline concentrations increased between baseline and t=0 min ( *P* <0.001) and returned to baseline at t=60 min ( *P* =1.0), while cortisol concentrations did not change over time ( *P* =1.0). In CON, noradrenaline concentrations remained unchanged between baseline and t=0 min ( *P* =0.378), whereas cortisol concentrations decreased ( *P* <0.001). Noradrenaline concentrations in CON increased from baseline to t=60 ( *P* =0.017), while cortisol concentrations returned to baseline ( *P* =1.0). At t=0 min, noradrenaline and cortisol concentrations were higher in HOT compared to CON ( *P* <0.05). At t=60 min, noradrenaline concentrations were lower in HOT compared to CON ( *P* =0.020), while cortisol concentrations did not differ between HOT and CON ( *P* =0.64). During CON, a positive correlation was found between glucose iAUC and cortisol concentrations at t=0 (Pearson’s *r* =0.617; *P=* 0.033). Furthermore, during HOT, a tendency towards a positive correlation was observed between glucose iAUC and noradrenaline concentrations at t=0 min (Pearson’s *r=* 0.560; *P* =0.058, **Supplementary Figure 3** ).

### Body mass

A significant time×condition interaction was observed for body mass ( *P* <0.001). During HOT, body mass decreased from 82.7±12.1 to 82.4±12.1 kg between baseline and t=0 min ( *P* <0.001) and did not return to baseline values at t=120 (82.5±12.1 kg; *P* =0.022). During CON, body mass was 83.2±12.4 kg at baseline and did not change throughout the test day.

### Dietary intake

During the 2 days prior to HOT and CON, no differences were observed in average daily intake of dietary energy (84±24 vs. 80±20 kJ/kg/d; *P=* 0.52), carbohydrate (43±4 vs. 44±7 En%; *P* =0.58), protein (18±3 vs. 19±3 En%; *P* =0.26) and fat (39±5 vs. 37±6 En%; *P* =0.28).

## Discussion

The present study shows a greater postprandial rise in circulating plasma glucose concentrations following glucose ingestion after a single session of infrared sauna bathing compared to a thermoneutral condition in individuals with T2DM, with no differences in circulating plasma insulin concentrations. In contrast to our hypothesis, a single session of infrared sauna bathing did not result in lower blood glucose excursions following glucose ingestion in individuals with T2DM.

In line with previous work on passive heat treatment 15 17 26 27 28 29 30 31 , we showed that infrared sauna bathing increased tympanic temperature, skin temperature, and heart rate ( Fig. 2a–c ). These physiological changes, though varying in magnitude, show some similarities to those elicited by low-intensity exercise 6 . It has been well established that exercise increases skeletal muscle blood flow, which partly contributes to increased glucose uptake in people with T2DM 3 7 . Given that infrared sauna bathing may also potently stimulate skeletal muscle blood flow, we hypothesized that a single session of infrared sauna bathing lowers glucose excursions during a subsequent OGTT in adults with T2DM. However, we observed a more (instead of less) pronounced postprandial rise in circulating plasma glucose concentrations, with no changes in insulin concentrations and insulin sensitivity, during the 2-h OGTT that was performed immediately following infrared sauna bathing when compared to the same treatment in a thermoneutral condition ( Fig. 3 **a** , [Fig FI02-2024-0041-dia-0003] **c** and 4 , respectively). Our data are in contrast with previous studies showing no impact of a single session of passive heat treatment (i. e., hot water immersion) on postprandial glucose concentrations and/or insulin sensitivity in individuals with T2DM 15 16 17 . However, it is important to note that previous studies in non-diabetic populations have also observed increased (rather than reduced) postprandial glucose concentrations with acute passive heat treatment compared to thermoneutral control settings 28 29 30 32 33 . Taken together, our study adds to the existing literature suggesting that a single session of passive heat treatment does not facilitate a reduction in postprandial glucose concentrations or an improvement in insulin sensitivity 15 16 17 28 29 30 32 33 .

Several mechanisms can be explored to elucidate the observed increase, instead of a decrease, in post-prandial rise in circulating plasma glucose concentrations. We explored whether a decline in blood volume through sweating might explain the more elevated post-prandial blood glucose concentrations following passive heat treatment. Participants lost 300±100 mL of body mass following passive heating, while no decline in body mass was observed in the thermoneutral condition. However, no significant changes were observed in blood hematocrit levels over time or between treatments. In line with previous work investigating the impact of hypohydration on glycemic excursions 34 , we did not detect any differences in plasma glucose data when data were corrected for blood sample hematocrit values. The initial elevation in plasma glucose concentrations may also be (partly) attributed to arterialization of venous blood samples after heating 35 36 . Nevertheless, the arterialization effect of local hand heating has been shown to disappear within ~15 min after removing the heat source 37 . How long this effect persists after whole-body heating is not known, though it is unlikely that arterialization explains the prolonged elevation in glucose concentrations observed up until 2 h after exiting the sauna.

As skin and tympanic temperature and heart rate increased, we reasonably assumed that peripheral blood flow increased following passive heat treatment 9 . However, enhanced peripheral blood flow did not result in an increase in peripheral blood glucose uptake, or was insufficient to attenuate post-prandial blood glucose excursions. Therefore, we speculate that increased muscle perfusion during heat stress primarily facilitates heat dissipation to the skin, rather than improving peripheral glucose uptake in skeletal muscle. Furthermore, heat stress-induced release of stress hormones may have stimulated hepatic glucose output, leading to an elevation in endogenous glucose appearance 9 31 38 39 . In accordance, we observed an elevation in plasma noradrenaline and cortisol concentrations measured immediately following infrared sauna bathing and a tendency towards a positive correlation between glucose iAUC and noradrenalin concentrations after infrared sauna bathing. This finding implies that a systemic stress response may have contributed to the observed elevation in postprandial blood glucose concentrations in this study. Finally, during heat stress, blood may have been redirected from the splanchnic region to accommodate increased skin perfusion 40 , potentially slowing gastric emptying and, as such, glucose absorption. Speculatively, this may also account for the prolonged postprandial elevation of blood glucose concentration.

Although a relatively small sample size was included, the robust cross-over study design allowed us to reliably detect relevant differences in body and skin temperature between the two conditions. Unfortunately, we were not able to measure rectal temperature and/or skin temperature at multiple body sites to provide a more accurate assessment of body temperature during and following passive heat treatment. Also, composite indicators of whole-body insulin sensitivity are not as reliable as measurements using the gold standard hyperinsulinemic-euglycemic clamp and glucose tracers. Therefore, to understand glucose fluxes and insulin action following heat treatment, future work that directly measures glucose fluxes is warranted. The present study should be regarded as a proof-of-principle, as the study design applied in the present study does not apply to normal, daily life conditions. Participants ingested 75 g glucose within 5 min for the OGTT following the passive heat treatment and control condition. The administration of such high glucose loads result in more rapid glucose absorption compared to the gradual absorption of glucose following ingestion of a normal mixed meal 41 . The timing of heat treatment, however, does not seem to impact subsequent post-prandial blood glucose responses. To illustrate, no difference was observed between glycemic excursions when performing the OGTT either during or 30 min after hot water immersion in individuals with T2DM 17 . Moreover, glycemic excursions did not differ 1 h 15 or 24 h 16 after hot water immersion in individuals with T2DM.

Interestingly, in contrast to the outcomes of most studies addressing the acute impact of heat treatment, prolonged passive heat treatment has consistently been found to improve glycemic excursions in healthy 42 , overweight 26 43 , and individuals with T2DM 44 . It seems that the thermal stress through repeated acute passive heating triggers adaptations that may improve thermoregulation in challenging environments. At present, the effects of prolonged application of frequent infrared sauna bathing on glycemic outcomes in T2DM remain to be investigated.

In conclusion, a single session of infrared sauna bathing does not attenuate the postprandial rise in circulating blood glucose concentrations during a subsequent OGTT in individuals with T2DM. Future work should focus on identifying underlying mechanisms by directly measuring glucose fluxes, investigating hormonal influences, and analyzing blood flow distribution to further explore the effects of different modalities of acute and more chronic passive heat treatment on glycemic excursions and cardiovascular risk factors in individuals with T2DM.
