# Supplementary material for: A Single Sauna Session Does Not Improve Postprandial Blood Glucose Handling in Individuals with Type 2 Diabetes Mellitus: A Cross-Over, Randomized, Controlled Trial
Source: Exp Clin Endocrinol Diabetes. 2024 Sep 26;132(11):622–30. doi: 10.1055/a-2406-4491 (PMC11560331; doi:10.1055/a-2406-4491)
Supplement: Supplementary file 1 — Supplementary Material [file 10-1055-a-2406-4491-02-2024-0041-dia.pdf]

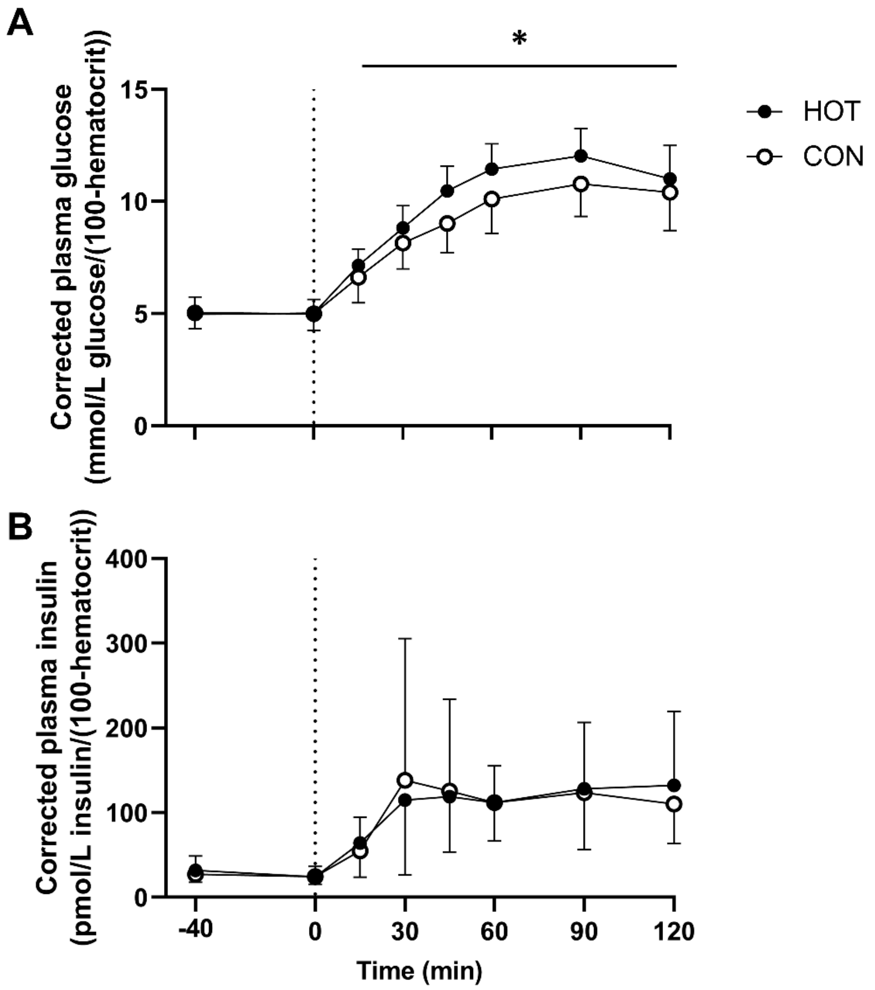


**Supplementary Figure 1.** Plasma glucose (A) and plasma insulin (B) concentrations corrected for haematocrit throughout the experimental visit. The glucose beverage was ingested at t=0 min (dotted line). Data are presented as means ± SDs, n=12. *HOT is significantly higher than CON (P<0.05). Data were analysed using two-way (time × condition) repeated measures ANOVAs. CON, control: 40 min at 21°C; HOT, infrared sauna: 40 min at 60°C.

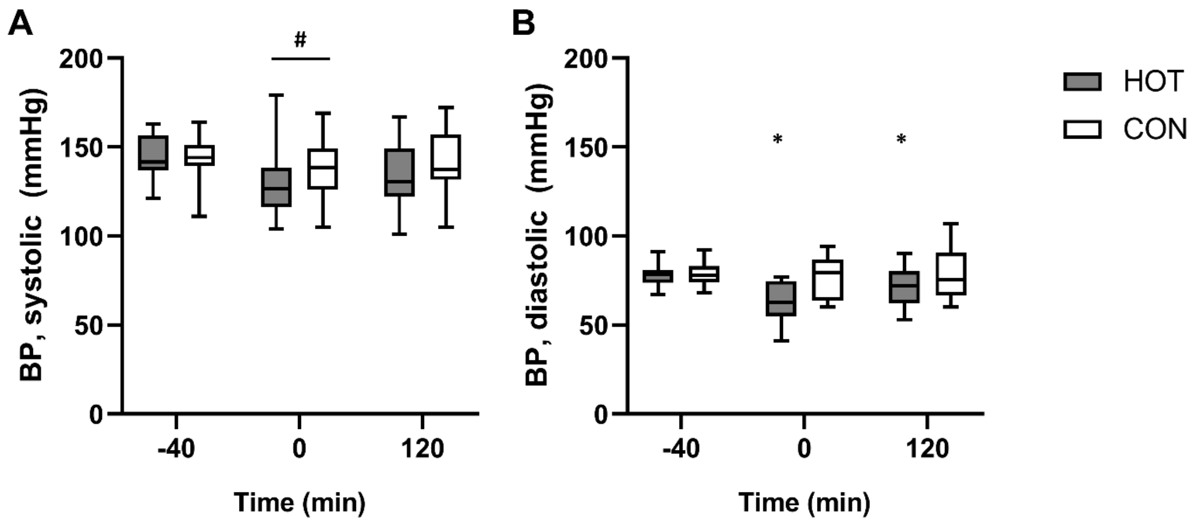


**Supplementary Figure 2.** Systolic (A) and diastolic (B) blood pressure at t=-40, 0, and 120 min. Data are presented in boxplots, in which whiskers represent minimum and maximum values, boxes range from the25th to the 75th percentile and the horizontal line represent the medians of the datasets, n=12.#Significantly lower than t=-40 (P<0.05). *HOT significantly lower than CON (P<0.05). Data were analysed using two-way (time × condition) repeated measures ANOVAs. BP, blood pressure; CON, control: 40 min at21°C; DBP, diastolic blood pressure; HOT, infrared sauna: 40 min at 60°C; SBP, systolic blood pressure.

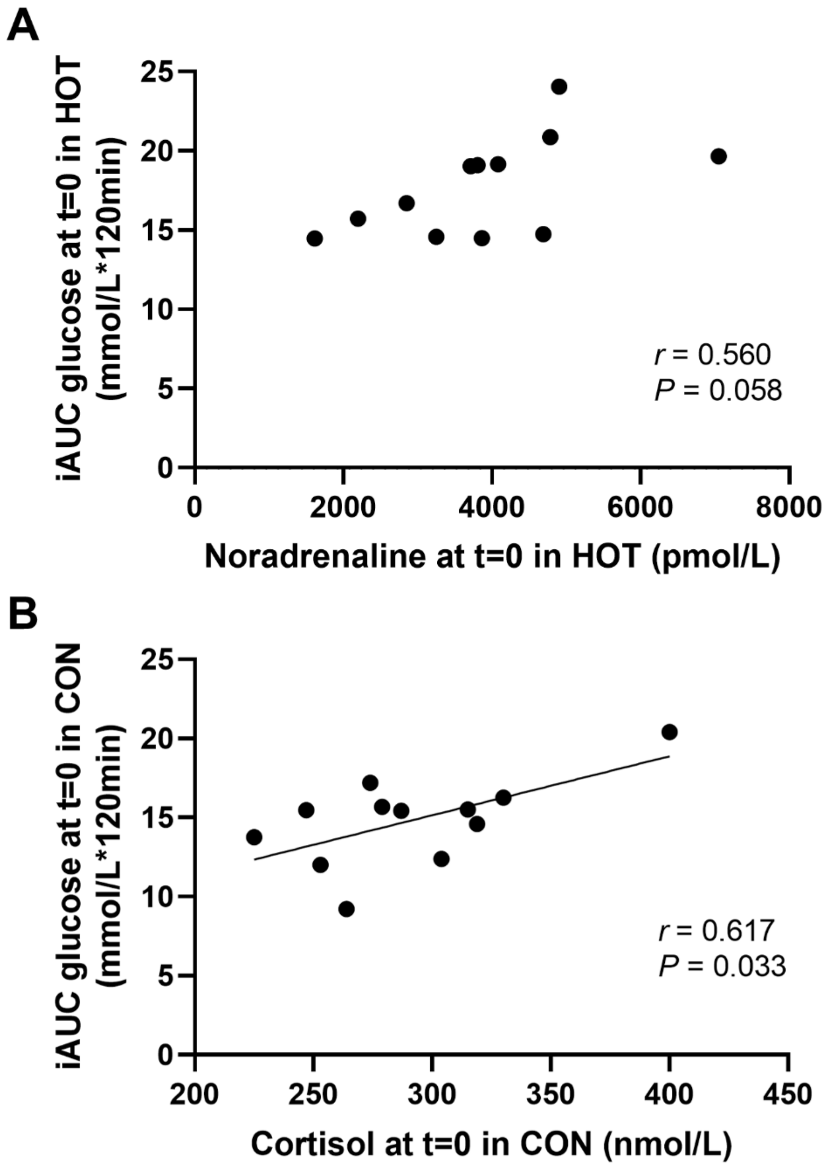


**Supplementary Figure 3.** Correlation between iAUC for glucose and noradrenaline concentrations at t=0 in HOT (A) and correlation between iAUC for glucose and cortisol concentrations at t=0 in CON (B). Individual data points are depicted, n=12. Pearson's correlation coefficients were determined to assess correlations. CON, control: 40 min at 21°C; HOT, infrared sauna: 40 min at 60°C; iAUC, incremental area under the curve.
